# Identifying the communication of burnout syndrome on the Twitter platform from the individual, organizational, and environmental perspective

**DOI:** 10.3389/fpsyg.2023.1236491

**Published:** 2023-10-19

**Authors:** Gabriela Ježková Petrů, Kristýna Zychová, Kateřina Drahotová, Kateřina Kuralová, Lucie Kvasničková Stanislavská, Ladislav Pilař

**Affiliations:** Department of Management, Faculty of Economics and Management, Czech University of Life Sciences Prague, Prague, Czechia

**Keywords:** burnout, communication, environmental factors, human resources management, individual factors, organizational factors, social media analysis, Twitter

## Abstract

Addressing the escalating prevalence of burnout syndrome, which affects individuals across various professions and domains, is becoming increasingly imperative due to its profound impact on personal and professional aspects of employees’ lives. This paper explores the intersection of burnout syndrome and human resource management, recognizing employees as the primary assets of organizations. It emphasizes the growing importance of nurturing employee well-being, care, and work-life balance from a human resource management standpoint. Employing social media analysis, this study delves into Twitter-based discourse on burnout syndrome, categorizing communication into three distinct dimensions: individual, organizational, and environmental. This innovative approach provides fresh insights into interpreting burnout syndrome discourse through big data analysis within social network analysis. The methodology deployed in this study was predicated upon the enhanced Social Media Analysis based on Hashtag Research framework and frequency, topic and visual analysis were conducted. The investigation encompasses Twitter communication from January 1st, 2019, to July 31st, 2022, comprising a dataset of 190,770 tweets. Notably, the study identifies the most frequently used hashtags related to burnout syndrome, with #stress and #mentalhealth leading the discussion, followed closely by #selfcare, #wellbeing, and #healthcare. Moreover, a comprehensive analysis unveils seven predominant topics within the discourse on burnout syndrome: organization, healthcare, communication, stress and therapy, time, symptoms, and leadership. This study underscores the evolving landscape of burnout syndrome communication and its multifaceted implications for individuals, organizations, and the broader environment, shedding light on the pressing need for proactive interventions. In organizations at all levels of management, the concept of burnout should be included in the value philosophy of organizations and should focus on organizational aspects, working hours and work-life balance for a healthier working environment and well-being of employees at all levels of management.

## 1. Introduction

Burnout syndrome has long been an increasingly discussed topic, affecting more professions and fields. While psychology primarily addresses its solution and prevention, it is crucial to recognize that prevention requires a multidisciplinary approach. Therefore, human resource management (HRM) must address this issue as well. From the original conception by Freudenberger (1974), who defined burnout as a state of physical exhaustion resulting from an individual’s professional activity, it is evident that the connection to HRM is relevant. Burnout syndrome is characterized by signs and symptoms stemming from complete energy depletion and deficiencies in resources or strength (Correia et al., 2021).

From HRM perspective, it is essential to note that specific jobs and job roles have a higher prevalence of burnout syndrome associated with elevated stress levels. Numerous studies have examined this correlation. Golonka et al. (2019) also highlight that burnout is linked to overlapping effects of other syndromes and disorders, such as depression and anxiety. Additionally, individual characteristics like neuroticism contribute to the propensity for burnout. Whether the burnout syndrome is independent remains unclear (Kurzthaler et al., 2017). However, as Bedrnová (2012) pointed out regarding HRM, we must acknowledge that burnout syndrome is not solely caused by emotional exhaustion, which can also stem from personal life issues. It is also characterized by depersonalization, feelings of alienation or detachment from oneself, and low personal achievements. Employee development plans, performance appraisals, workplace relationships, employee support, and perceptions of job and role can all be linked to burnout syndrome (Bedrnová, 2012).

Burnout is a complex phenomenon influenced by various factors at different levels. Understanding the causes of burnout can help organizations and individuals develop effective interventions and prevention strategies (McCormack and Cotter, 2013; Green et al., 2014). Therefore, creating a work environment that promotes employee well-being, provides adequate resources and social support, and recognizes and values employee contributions is essential. Understanding factors affecting burnout is vital to care employees’ psychosocial well-being, organizational effectiveness, and consent health (Maslach, 2003). According to Maslach (2003), future studies should examine additional factors at the client, provider, and organizational level variables to determine their relative impact on burnout.

Pilař et al. (2021) recognize significant research potential in the data generated through active and passive digital traces left by social network users, for whom this form of communication has become an integral part of their daily lives. People’s communication on social media, and consequently the content of the data conveyed through these platforms, encompass a wide range of facts, opinions, ideas, and emotions (Steinert and Dennis, 2022). The influence of emotion and the concept of feedback expectancy on social networking has been addressed by Stsiampkouskaya et al. (2021). In their research, Burbach et al. (2020) investigated whether or not a user of online social networks disseminates information and found that the type of network has only a weak influence on the dissemination of content, while the kind of message has an evident effect on how many users receive the message. Radwan (2022) addressed the topic of cultural identity (the set of memories, impressions, ideologies, ideas, etc.), that maintain people’s civilizational identity. His research focused on recognizing the influence of social media on selected components of cultural identity demonstrated a high rate of change in cultural identity with social media use. Stieglitz and Dang-Xuan (2013) point out that this vast data collection provides valuable grounds for communication analysis and research. In line with the growing interest in social media communication, this study explicitly investigates the communication surrounding burnout syndrome from the perspective of the Twitter platform users.

In today’s digital era, where social media has emerged as a primary mode of communication and emotional expression, it becomes imperative to comprehend how individuals communicate about critical topics, such as burnout syndrome. Although numerous studies focus on burnout in psychology and HRM (e. g. Schaufeli et al., 2009; Maslach and Leiter, 2016; Russell et al., 2020; González-Rico et al., 2022; Storti et al., 2023), only some delve deeply into the discourse surrounding burnout on platforms like Twitter (Harren et al., 2021; Black et al., 2022). This study offers a novel insight into the communication dynamics of burnout syndrome by investigating how individuals, organizations, and broader societal contexts articulate this phenomenon on Twitter. Recognizing these communication patterns can empower organizations to understand better and address their employees’ needs and may serve as a foundation for future preventive strategies. The growing importance of addressing burnout in today’s fast-paced, digitally connected world makes it a compelling issue deserving of our attention. Given the rising prevalence of burnout across various professions and sectors, this study is timely and essential.

This paper aims to identify communication focused on burnout syndrome on the Twitter from the perspective of individual, organizational and environmental factors, bringing a new perspective to the burnout syndrome communication research field.

This study addresses several key questions: How do the Twitter users discuss the topic of burnout and which hashtags do they employ when communicating about burnout? What is the frequency of the hashtag usage concerning burnout? Which topics are most discussed in the context of burnout syndrome on the Twitter? To which factors affecting burnout syndrome do the communicated hashtags relate? Lastly, how does this communication on the Twitter impact HRM and employee well-being?

Thus, our study extends beyond the confines of social media, seeking to understand how communication on Twitter can influence HRM practices and, consequently, employee well-being. In this way, we strive to provide actionable insights that can lead to more effective strategies for combating burnout and enhancing the quality of life in contemporary workplaces. In light of these considerations, our study is a pertinent exploration into the world of burnout syndrome communication on Twitter, offering valuable insights and implications for researchers, practitioners, and individuals alike.

## 2. Theoretical background

Edú-Valsania et al. (2022) reported that Graham Greene was the first author to use the term burnout in his novel “*A Burnt-Out Case*,” published in 1960. Subsequently, the term was introduced into the psychological domain by Freudenberger (1974), who described burnout as a state of exhaustion, fatigue, and frustration resulting from professional activities that fail to yield anticipated outcomes. Maslach (1976) further contributed to the understanding of burnout, defining it as a gradual process of fatigue, cynicism, and diminished commitment among social service employees. Later, Maslach and Jackson (1981) defined burnout as a psychological syndrome characterized by emotional exhaustion, depersonalization, and reduced professional efficacy. According to Edú-Valsania et al. (2022), it is crucial to conceptualize burnout as a syndrome, wherein a syndrome is understood as a collection of symptoms and signs that coexist and clinically define a particular condition separate from others. In their study, Edú-Valsania et al. (2022) provide a comprehensive overview of the most up-to-date and empirically supported explanatory theories of burnout. Specifically, the paper details the following theories: (1) social cognitive theory, (2) social exchange theory, (3) organizational theory, (4) structural theory, (5) job demands-resources theory, and (6) emotional contagion theory.

While consensus is yet to be reached regarding the conceptualization and measurement of burnout, there is some agreement that it is not a one-dimensional occupational phenomenon and that exhaustion represents a core component of burnout (Demerouti and Bakker, 2022). One widely recognized tool for assessing burnout syndrome is the Copenhagen Burnout Inventory (CBI), which evaluates personal, work-related, and client-related burnout across various job domains. The CBI defines burnout as the state of physical and mental burnout and exhaustion, specifically focusing on the burnout phenomenon itself (Kristensen et al., 2005).

### 2.1. Types of burnout

*Personal burnout* refers to how individuals experience physical and psychological fatigue and exhaustion, irrespective of their occupational status. The Copenhagen Burnout Inventory (CBI) includes a subscale measuring personal burnout. This 6-item subscale comprises questions such as “*How often do you feel tired?*,” “*How often do you think: ‘I cannot take it anymore’*” and “*How often do you feel worn out?*” (Kristensen et al., 2005, p. 200). Notably, leaders play a multifaceted role in fostering resilience within organizations. They should personally invest in resources that enhance their strength to mitigate the risk of personal burnout (McEwen, 2022). Another type of burnout (one part of CBI) is *work-related burnout*. The work-related burnout has also been identified as a contributing factor to absence from work due to long-term illness and, thus, the loss of productivity (Hallsten et al., 2011). Work-related burnout is common and detrimental to employees in many industries (Lam et al., 2022). In addition, work-related burnout resulting from chronic stress is well-documented (Maslach and Jackson, 1984; Kristensen et al., 2005; Shirom et al., 2009; Gray-Stanley and Muramatsu, 2011; Maslach and Leiter, 2016; Wood et al., 2020). The third type of burnout is CBI’s *client-related burnout* category, referring to the degree of physical and psychological fatigue and exhaustion that a person perceives concerning work with clients (Kristensen et al., 2005). The client is a general term covering people such as patients, students, teachers, children, etc., who receive service (i.e., service recipients) from people who provide the service (i.e., service providers). The items of client-related burnout precisely assess the connection between fatigue and people-centered work (Chin et al., 2018).

High emotional exhaustion, cynical attitudes, and a diminished sense of personal accomplishment at work characterize *professional burnout*. Recent changes in healthcare delivery have also raised concerns that provider burnout may worsen if increased patient care and administrative demands outpace resources (Shanafelt et al., 2015). Indeed, according to a national study by Shanafelt et al. (2015), burnout among physicians compared to the general population increases. Over the past two decades, a growing body of evidence has suggested that personality may significantly contribute to the development of burnout (Alarcon et al., 2009). Further, Jin et al. (2015), in their study on teachers, identified teacher personality as one of the most influential predictors of burnout.

*Occupational burnout* can have adverse consequences not only at an individual level (e.g., physical and mental health problems) (Suñer-Soler et al., 2013) but also at an organizational level (e.g., absenteeism, poor performance at work, misjudgments and errors, job turnover) (Ochoa, 2018). From an individual and an organizational perspective, preventing occupational burnout has been viewed as the best approach to deal with this phenomenon (Borysiewicz, 2010). In addition, according to Žutautienė et al. (2020), primary prevention of occupational burnout should be considered a public health priority worldwide.

Chronic stress can lead to burnout in professions that involve working with people. Burnout syndrome is characterized by persistent physical and emotional exhaustion and behavioral issues (Suyi et al., 2017). Workplace behavior changes may be the first noticeable signs in a team or collective. However, the effects of burnout extend beyond individual behavior and can be observed at the organizational level, impacting employee performance, communication, teamwork, and meeting deadlines. In addition to stressors, burnout is influenced by demographic factors and the level of social support. Several studies have addressed social support as a preventive measure against burnout syndrome (Halbesleben, 2006; González-Morales et al., 2012). These studies have highlighted the organizational dimension of burnout, with employees closely perceiving how burnout affects their colleagues, emphasizing the crucial role of the work environment in its development. HRM has recently highlighted the quality of working relationships and employee care. Research indicates that certain professions, such as healthcare, are more susceptible to burnout syndrome (Manna et al., 2022).

The concept of quality has expanded to include the non-work component of life, leading to the emergence of the work-life balance theory and various theoretical concepts that consider both work and personal aspects of life (Agrawal et al., 2022). Work-life quality comprises two fundamental components: achievement and positive experiences (enjoyment). These components counterbalance to negative influences associated with burnout syndrome (Aloulou et al., 2023). In the 1980s, Freudenberger and Torkelsen (1984); Freudenberger and North (1985) described the progression of burnout syndrome. The incubation period for burnout cannot be uniformly defined as it depends on various external circumstances and individual personality factors (Amir et al., 2018).

### 2.2. The multifaceted and complex causes of burnout

**Figure 1:**
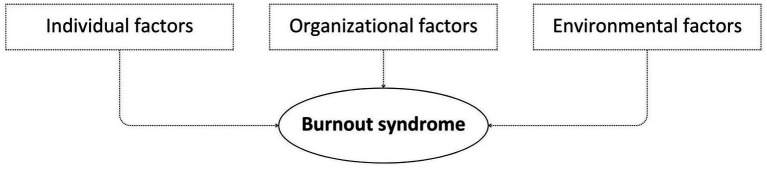
The multifaceted and complex causes of burnout: interplay of individual, organizational, and environmental factors. Source: Own elaboration (2023).

Burnout syndrome is a prevalent problem that affects many employees, especially those in high-stress jobs. Burnout can lead to reduced job satisfaction, decreased productivity, and increased absenteeism, harming individual and organizational performance (Maslach and Leiter, 2016). The causes of burnout are multifaceted and complex, which involve a combination of individual, organizational, and environmental factors (McCormack and Cotter, 2013; Green et al., 2014; Galletta et al., 2016), as shown in the Figure 1.

*Individual factors* such as personality traits, coping skills, and work style have been found to contribute to burnout (Hakanen et al., 2006; McCormack and Cotter, 2013; Green et al., 2014; Rees et al., 2019). According to Maslach and Leiter (2016), employees with a high workload, long hours, and little control over their work are more likely to experience burnout. Thus, low levels of job autonomy, increased work demands, and job insecurity are more susceptible to burnout (Hakanen et al., 2006). One significant cause of burnout is a work-life imbalance, which arises when work demands interfere with the employee’s personal life (Greenhaus and Allen, 2011). Greenhaus and Allen (2011) pointed out that employees with long commuting times, inadequate childcare, or other family responsibilities may experience difficulties balancing their work and personal life, leading to stress and burnout.

*Organizational factors* such as inadequate resources, job control, social support, and low levels of job satisfaction have also been linked to burnout (McCormack and Cotter, 2013; Green et al., 2014; Maslach and Leiter, 2016). Maslach and Leiter (2016) pointed out that a lack of job control, inadequate resources, and poor communication can lead to chronic stress and burnout. Bakker et al. (2014) have found that employees who work in organizations with high job demands and low levels of social support are more likely to experience burnout. Research has also found that employees who do not receive adequate recognition or support from their supervisors are more likely to experience burnout (Halbesleben and Buckley, 2004). According to Hakanen et al. (2006), lacking organizational support, such as inadequate resources, low pay, and job insecurity, can lead to burnout.

Additionally, a lack of recognition, feedback, and opportunities for growth can contribute to feelings of depersonalization and emotional exhaustion (Maslach and Leiter, 2016). Bakker et al. (2014) stressed that employees who feel isolated, disconnected, or unsupported by their colleagues or supervisors may experience high levels of burnout. The lack of control or autonomy in the workplace can lead to frustration, helplessness, and reduced job satisfaction (Maslach and Leiter, 2016). Moreover, work-related conflict and incivility (such as bullying, harassment, and discrimination) can also contribute to burnout (Cortina et al., 2013).

*Environmental factors* can also contribute to burnout. For instance, changes in the work environment, such as restructuring, downsizing, or mergers, can cause uncertainty and ambiguity, leading to increased job stress and burnout (Brough et al., 2005; McCormack and Cotter, 2013; Galletta et al., 2016; Rees et al., 2019). In addition, economic instability, political turmoil, and natural disasters can also have significant effects (Shanafelt et al., 2020). As Shanafelt et al. (2020) pointed out, employees who work in high-risk or hazardous environments, such as healthcare employees, firefighters, or law enforcement officers, may experience chronic stress and burnout due to exposure to traumatic events and emotional strain. For example, healthcare employees during the COVID-19 pandemic have experienced high levels of burnout due to the stress of working in a high-risk environment and the emotional toll of caring for sick patients (Shanafelt et al., 2020). Further, the blurring of boundaries between work and personal life due to technological advances and the increasing use of information and communication technology can lead to work overload and difficulties achieving a work-life balance, resulting in burnout (Hakanen et al., 2006). Clarifying our understanding of environmental factors associated with burnout can help agencies and leaders work to effectively implement organizational interventions (Green et al., 2014).

## 3. Materials and methods

**Figure 2:**
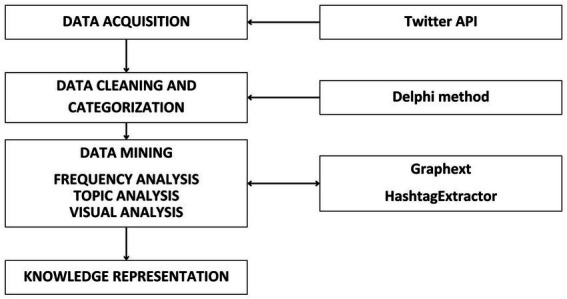
Four steps of the social media analysis based on the SMAHR framework for the hashtag research. Source: Own elaboration (2023).

The methodology deployed in this study was predicated upon the enhanced Social Media Analysis based on Hashtag Research (SMAHR) framework as outlined by Pilař et al. (2021). This SMAHR framework-driven analytical procedure encompassed four distinct stages, as depicted in the Figure 2.

### 3.1. Data acquisition

The primary objective of this stage was to collect data from the Twitter featuring the hashtag #burnout. The timeframe for this data collection spanned from January 1st, 2019, to July 31st, 2022, with the Twitter Application Programming Interface (API) serving as the mechanism for extraction (Platform Developer, 2022). The Twitter API v2 was utilized to garner the data in question, facilitated by the Tractor software (Tractor, 2023). This software was specifically employed to download tweets encapsulating the hashtag #burnout. A substantial accumulation of 190,770 tweets from 71,873 unique Twitter users was extracted throughout this period. The dataset compiled encompasses the entirety of tweets, inscribed with the hashtag #burnout, posted by users on the Twitter within the predefined observational period.

### 3.2. Data cleaning and categorization

In the initial phase, the data was subjected to a cleaning process. Out of the 76,971 unique hashtags associated with the #burnout hashtag, the 80 most frequently used ones were chosen for further analysis (see Table 1). However, the 14 hashtags were excluded based on their content, explicitly removing illogical or unclear ones (see Table 2 for more details).

**Table 1:** The frequency of the hashtags related to burnout syndrome on the Twitter.

Position	Hashtag	Total count
1	#stress	16,889
2	#mentalhealth	13,306
3	#selfcare	5,864
4	#wellbeing	5,345
5	#healthcare	5,131
6	#leadership	4,782
7	#depression	4,441
8	#wellness	4,328
9	#covid19	3,818
10	#anxiety	3,335
11	#worklifebalance	3,314
12	#hr	3,223
13	#health	3,015
14	#work	2,881
15	#productivity	2,596
16	#mindfulness	2,242
17	#resilience	2,198
18	#podcast	2,175
19	#wfh	1,929
20	#physician	1,885
21	#energy	1,872
22	#medtwitter	1,868
23	#boundaries	1,860
24	#healing	1,802
25	#pandemic	1,772
26	#stressmanagement	1,747
27	#depressionen	1,734
28	#recovery	1,694
29	#breakfastleadership	1,615
30	#business	1,587
31	#workplace	1,584
32	#remotework	1,479
33	#millennials	1,465
34	#management	1,461
35	#naturalhealing	1,408
36	#career	1,365
37	#mentalhealthmatters	1,301
38	#motivation	1,268
39	#travail	1,246
40	#physicians	1,240
41	#coaching	1,219
42	#mentalhealthawareness	1,205
43	#medicine	1,195
44	#chi	1,178
45	#ai	1,136
46	#meded	1,128
47	#nurses	1,102
48	#burnoutprevention	1,085
49	#fatigue	1,077
50	#bĂĽcher	1,071
51	#geschenk	1,057
52	#talento	1,050
53	#physicianburnout	1,046
54	#reclutamiento	1,043
55	#balance	1,015
56	#felicidad	1,014
57	#vida	1,012
58	#competitividad	1,000
59	#marcapersonal	993
60	#innovaciĂłn	971
61	#covid	939
62	#entrepreneur	931
63	#workfromhome	914
64	#qvt	910
65	#employees	867
66	#caregiver	854
67	#futureofwork	827
68	#doctors	814
69	#ehr	814
70	#success	799
71	#leaders	769
72	#salud	768
73	#mindset	753
74	#santĂ©	752
75	#stressrelief	732
76	#exhaustion	697
77	#life	695
78	#culture	691
79	#employeewellbeing	690
80	#selflove	490

Source: Own elaboration based on collected data from the Twitter (2023).

**Table 2:** Removed hashtags.

Position	Hashtag	Total count
44	#chi	1,178
50	#bĂĽcher	1,071
51	#geschenk	1,057
52	#talento	1,050
54	#reclutamiento	1,043
56	#felicidad	1,014
57	#vida	1,012
58	#competitividad	1,000
59	#marcapersonal	993
60	#innovaciĂłn	971
64	#qvt	910
69	#ehr	814
72	#salud	768
74	#santĂ©	752

Source: Own elaboration (2023).

After the cleaning phase, the categorization of the 66 remaining hashtags was realized. The Delphi method was used to categorize the hashtags and provide a new perspective from expert HRM practitioners on the various aspects influencing burnout syndrome. It is a collaborative approach that facilitates structured and anonymous communication among experts to reach a consensus (Brady, 2015). This approach’s essential advantage is avoiding confrontation between the experts (Okoli and Pawlowski, 2004). Three independent HRM experts participated in the categorization and classified the hashtags into individual, organizational, and environmental factors. Another HRM expert subsequently reviewed the results, incorporating feedback into the analysis, leading to further discussions and finalizing the results.

### 3.3. Data mining

This stage’s objective is to extract pertinent and valuable information from the abundant data produced by users across various social media platforms.

#### 3.3.1. Frequency analysis

This analysis is intended to pinpoint the hashtags most frequently used. By leveraging the Hashtag Extractor software (Pilař et al., 2021), all the hashtags were extracted from individual Tweets. The count of the particular hashtags was computed by importing them into the Gephi 0.9.2 software (Bastian et al., 2009).

#### 3.3.2. Topic analysis

This method is utilized to discern the principal subjects or themes conveyed within a large dataset, such as posts on social media platforms. In intricate networks, like those of social media, specific nodes (e.g., hashtags or words) exhibit more interconnectedness among themselves than with the remaining components of the network. It is feasible to discern topics based on the clusters of the particular hashtags. The objective of this phase was to delineate the thematic structure of discussions related to burnout on the Twitter. Unlike frequency analysis, topic analysis was based on complete tweets (not merely hashtags). Graphext software (Graphext, 2023) was employed for this analysis. To dissect the community structure of our network, Graphext applied a modified variant of the Louvain algorithm (Blondel et al., 2008). The network was established based on the interconnectivity of individual words within the tweet. The Louvain algorithm implements an iterative procedure of allocating nodes to clusters to optimize a performance metric known as modularity. This metric measures the comparative density of edges within clusters as opposed to those between clusters. The quantity of distinct communities within the dataset was determined as follows:


$$\Delta Q=\left[\frac{\sum_{in}+2k_{i,in}}{2m}-{\left(\frac{\sum_{tot}+k_{i}}{2m}\right)}^{2}\right]-\left[\frac{\;\sum_{in}}{2m}-{\left(\frac{\sum_{tot}}{2m}\right)}^{2}\right]$$

where $\underset{in}{\sum }$ is the sum of weighted links inside the community, $\underset{tot}{\sum }$ is the total number of weighted connections inside the community, $k_{i}$ is the total number of weighted links related to the community hashtags, and i , $k_{i,in}$ is the total weighted linkages from an individual to the community hashtags, and m is the normalization factor, calculated as the total weighted links over the entire graph (Blondel et al., 2008).

#### 3.3.3. Visual analysis

Techniques for network visualization, such as force-directed layouts, can emphasize various facets of a network, including the density of connections or topic polarization. This phase aimed to discern the polarity of the identified topics. Utilizing the ForceAtlas2 layout technique, a two-dimensional graph was fabricated for visual analysis. An enhanced variant of the ForceAtlas algorithm was deployed, dubbed ForceAtlas2, which concentrates on voluminous networks. This approach harnesses visual representations of smaller samples to pinpoint intercommunity connections within network communities (Jacomy et al., 2014). Visual analysis was executed using the Graphext software (Graphext, 2023).

### 3.4. Knowledge representation

Knowledge representation is a process that leverages visualization tools to elucidate the discoveries of data mining activities. It underscores the synthesis of individual values and outcomes from the data evaluation stage. The objective of this phase is to bring to the fore pivotal findings from prior analyses.

## 4. Results and discussion

Based on the collected data, the frequency analysis was conducted to examine the frequency of the hashtags concerning hashtag #burnout. As presented in the Table 3, the 66 hashtags were identified and analyzed.

**Table 3:** The frequency of the used hashtags related to burnout syndrome on the Twitter.

Position	Hashtag	Total count	Position	Hashtag	Total count
1	#stress	16,889	34	#management	1,461
2	#mentalhealth	13,306	35	#naturalhealing	1,408
3	#selfcare	5,864	36	#career	1,365
4	#wellbeing	5,345	37	#mentalhealthmatters	1,301
5	#healthcare	5,131	38	#motivation	1,268
6	#leadership	4,782	39	#travail	1,246
7	#depression	4,441	40	#physicians	1,240
8	#wellness	4,328	41	#coaching	1,219
9	#covid19	3,818	42	#mentalhealthawareness	1,205
10	#anxiety	3,335	43	#medicine	1,195
11	#worklifebalance	3,314	45	#ai	1,136
12	#hr	3,223	46	#meded	1,128
13	#health	3,015	47	#nurses	1,102
14	#work	2,881	48	#burnoutprevention	1,085
15	#productivity	2,596	49	#fatigue	1,077
16	#mindfulness	2,242	53	#physicianburnout	1,046
17	#resilience	2,198	55	#balance	1,015
18	#podcast	2,175	61	#covid	939
19	#wfh	1,929	62	#entrepreneur	931
20	#physician	1,885	63	#workfromhome	914
21	#energy	1,872	65	#employees	867
22	#medtwitter	1,868	66	#caregiver	854
23	#boundaries	1,860	67	#futureofwork	827
24	#healing	1,802	68	#doctors	814
25	#pandemic	1,772	70	#success	799
26	#stressmanagement	1,747	71	#leaders	769
27	#depressionen	1,734	73	#mindset	753
28	#recovery	1,694	75	#stressrelief	732
29	#breakfastleadership	1,615	76	#exhaustion	697
30	#business	1,587	77	#life	695
31	#workplace	1,584	78	#culture	691
32	#remotework	1,479	79	#employeewellbeing	690
33	#millennials	1,465	80	#selflove	490

Source: Own elaboration based on collected data from the Twitter (2023).

The Table 3 illustrates the distribution of the hashtags, highlighting their frequencies. The most prominent hashtag is #stress, which occurs 16,889 times—followed by #mentalhealth with 13,306 occurrences, #selfcare with 5,864 occurrences, and #wellbeing with 5,345 occurrences, respectively. In the subsequent analysis, these four most frequent hashtags, along with #depression in the 7th position, are classified under individual factors. The hashtag #healthcare appears in the 5th place with 5,131 occurrences, and due to its general nature, it is categorized as an environmental factor. Additionally, #covid19 ranks 9th, and #health ranks 13th, falling under environmental factors. Among the hashtags classified under organizational factors, the most prominent ones are #leadership in 6th place with 4,782 occurrences, #worklifebalance in 11th place with 3,314 occurrences, #hr. in 12th place with 3,223 occurrences, #work in 14th place, and #productivity in 15th place, both with 2,596 occurrences. Upon comparing the occurrence counts, it becomes evident that the hashtags related to individual factors, such as #stress, #mentalhealth, #selfcare, #wellbeing, and #depression, dominate the communication. The hashtags related to environmental factors, including #healthcare, #covid19, and #health, rank second in frequency, likely influenced by the ongoing COVID-19 pandemic. The third most frequent category of the hashtags pertains to work and working life, encompassing #leadership, #hr., #work, #wfh, and #stressmanagement.

A meticulous examination of the Twitter hashtags has determined that the hashtags related to burnout can be classified into three core groups: *individual*, *organizational*, and *environmental factors* (see Tables 4–6). This systematic categorization facilitates a holistic comprehension of the diverse elements that contribute to the occurrence of burnout and thus reflects people’s real-world communication about burnout syndrome.

**Table 4:** The hashtags shared alongside the hashtag #burnout categorized as individual factors.

Individual factors		
Position	Hashtag	Total count
1	#stress	16,889
2	#mentalhealth	13,306
3	#selfcare	5,864
4	#wellbeing	5,345
7	#depression	4,441
8	#wellness	4,328
10	#anxiety	3,335
11	#worklifebalance	3,314
15	#productivity	2,596
16	#mindfulness	2,242
17	#resilience	2,198
21	#energy	1,872
23	#boundaries	1,860
27	#depressionen	1,734
38	#motivation	1,268
42	#mentalhealthawareness	1,205
49	#fatigue	1,077
55	#balance	1,015
70	#success	799
73	#mindset	753
75	#stressrelief	732
76	#exhaustion	697
80	#selflove	490

Source: Own elaboration based on collected data from the Twitter (2023).

**Table 5:** The hashtags shared alongside the hashtag #burnout categorized as organizational factors.

Organizational factors		
Position	Hashtag	Total count
6	#leadership	4,782
12	#hr	3,223
14	#work	2,881
19	#wfh	1,929
26	#stressmanagement	1,747
29	#breakfastleadership	1,615
30	#business	1,587
31	#workplace	1,584
32	#remotework	1,479
33	#millennials	1,465
34	#management	1,461
36	#career	1,365
39	#travail	1,246
41	#coaching	1,219
48	#burnoutprevention	1,085
62	#entrepreneur	931
63	#workfromhome	914
65	#employees	867
67	#futureofwork	827
71	#leaders	769
79	#employeewellbeing	690

Source: Own elaboration based on collected data from the Twitter (2023).

**Table 6:** The hashtags shared alongside the hashtag #burnout categorized as environmental factors.

Environmental factors		
Position	Hashtag	Total count
5	#healthcare	5,131
9	#covid19	3,818
13	#health	3,015
18	#podcast	2,175
20	#physician	1,885
22	#medtwitter	1,868
24	#healing	1,802
25	#pandemic	1,772
28	#recovery	1,694
35	#naturalhealing	1,408
37	#mentalhealthmatters	1,301
40	#physicians	1,240
43	#medicine	1,195
45	#ai	1,136
46	#meded	1,128
47	#nurses	1,102
53	#physicianburnout	1,046
61	#covid	939
66	#caregiver	854
68	#doctors	814
77	#life	695
78	#culture	691

Source: Own elaboration based on collected data from the Twitter (2023).

The *individual factors* group related to burnout contains the 23 hashtags (see Table 4), which can be divided into five groups (mental health, work-life balance, coping skills, productivity, and personality traits). The most posted hashtags regarding individual burnout factors were #stress (16,899 occurrences) and #mentalhealth (13,306 occurrences). Wu and Hong (2022), in their analysis, suggested that 277 out of all 14,015 comments contained the following selected mental health-related words: depression, depressed, anxiety, anxious, stress, and disorder(s). This analysis was confirmed by Golonka et al. (2019). Thus, individual factors of burnout are anxiety, neuroticism, and depression. According to the analysis, #depression was in the 7th place (4,441 occurrences) and #depressionen in the 27th place (1,734 occurrences) and #anxiety in the 10th position (3,335 occurrences). Workplace conditions cause job stress and negatively impact personal performance and overall physical and mental health (Parker and DeCotiis, 1983). Research by Wu et al. (2021) showed that perceived life stress and job stress are predictors of occupational burnout and play a significant role. If ignored, stress can lead to burnout (Thomas et al., 2019). Research also supports the empirical overlap of emotional exhaustion and depression (Golonka et al., 2019). According to Maslach et al. (2001), burnout is a syndrome described as a state of exhaustion. Exhaustion is connected not only to burnout but also to fatigue. According to Sikaras et al. (2021), both fatigue and burnout can lead to a feeling of mental and physical exhaustion. Overall, according to Weston et al. (2019), a pilot evaluation of the mental health awareness for managers training suggests that courses on mental health awareness can positively impact attendees’ awareness of and confidence in dealing with mental health issues in the workplace. However, the #mentalhealthawareness was in the 42nd position (1,205 occurrences), strongly connected to burnout prevention.

Based on a review of research and an empirical study according to Sikaras et al. (2021), the link between anxiety and burnout, particularly the dimension of emotional exhaustion, has been confirmed. According to Golonka et al. (2019), among individual characteristics, depression is revealed to be the most important variable. In addition, their research showed that exhaustion is explained mainly by individual factors such as neuroticism and depression (Golonka et al., 2019). However, #exhaustion was posted very rarely (697 occurrences).

The #selfcare is among the most mentioned hashtags (3rd position, 5,864 occurrences) regarding individual burnout factors. A few recently carried out studies highlighted the relationship between burnout and self-care. One study is focused on doctors breaching the boundary between doctors’ personal and professional life is potentially a risk for burnout (Morris et al., 2023). Another research for nursing professionals demonstrated that an informal attempt at self-care, like the work processes associated with personal experiences, edifies informal strategies (Sonnentag, 2015). So, it is essential, therefore, the instrumentalization for self-care (Camargo et al., 2021). The #selflove (80th position, 490 occurrences) is not much associated with burnout in scientific articles. The topic of self-love in leadership was addressed by Maharaj and April (2017), who suggest that self-love is at the core of authenticity, servant leadership, empathy, caring for employees, and the ability to listen. Self-love is a rare but vital phenomenon that requires more scientifical understanding.

Another most posted was #wellbeing (5,345 occurrences). The research on well-being and burnout among healthcare professionals dealt with burnout measures to assess well-being (Eckleberry-Hunt et al., 2018). The term well-being is associated with the other hashtags #worklifebalance (3,314 occurrences), #wellness (4,328 occurrences), #energy (1,872 occurrences) and #balance (1,115 occurrences). According to a study by Neumann et al. (2018) focused on oncology nurses, work-life balance is associated with burnout. Thus, one-third of the oncology nurses feel that their work schedule does not provide enough time for personal or family life (Neumann et al., 2018). Keeping a healthy balance between the time and energy one devotes to a career and life outside of work is crucial to preventing burnout (Allen, 2019). So, burnout is considered an occupational disease expressed by loss of mental and physical energy due to prolonged and unsuccessful coping with stressors at work (Grossman et al., 2019).

On the other hand, wellness was revealed as a mitigating factor for burnout. According to Brasfield et al. (2019), wellness may relieve professional stress. Brasfield et al.’s (2019) research results indicate significant relationships between burnout and wellness indicators.

In 16th place was the hashtag #mindfulness (2,242 occurrences). The systematic review focused on healthcare professionals identified that mindfulness-based interventions reduced stress and burnout and increased self-compassion and general health (Sarazine et al., 2021). Other coping skills, #mindset (73rd position) and #stress relief (75th position) were placed in the last positions. However, Zarrinabadi et al.’s (2023) study investigated whether teachers’ mindsets about stability or malleability of teaching ability (fixed and growth mindsets) and self-efficacy predicted their burnout and professional identity beliefs. Work-related stress is a significant occupational health problem associated with adverse physical and mental health effects. Physical stress relief methods like yoga and massage therapy may reduce occupational stress (Zhang et al., 2021). Additionally, meditation and mindfulness have been used in many cultures as stress relief (Anderson et al., 1999).

Rapp et al. (2021) concluded that the contextual shock of the COVID-19 pandemic resulted in an increased incidence of boundary violations-undesired disruptions between work and other important life domains such as personal and family life. These boundary violations, which we classify as physical, temporal, or knowledge-based, frequently correspond to greater burnout reports manifested by exhaustion, detachment, and inefficacy. According to research by Graña et al. (2021), Pearson correlations showed that #motivation (38th position among the hashtags) is negatively related to burnout and positively to engagement, while burnout and engagement are inversely related.

In addition, the hashtag success is related to burnout in the context of productivity. Grebski and Mazur (2022) state that undetected and untreated burnout leads to decreased employee productivity. So, preventing employee burnout leads to increased productivity and economic success for the company. The hashtag #resilience as a personal trait was in the 17th position. It was the only one belonging to the group of individual factors. The connection between burnout and resilience was confirmed. According to the research of Győrffy (2019), results have shown that internal resources, coping strategies and resilience play a crucial role in burnout and its decrease.

The *organizational factors* group related to burnout contains the 21 hashtags (see Table 5). After further investigation, this group can be divided into four subgroups: work-related factors, telecommuting, personal development and well-being, and generational focus.

Work-related factors encompass the hashtags such as #work, #workplace, #business, #travail, #management, #leadership, #breakfastleadership, #leaders, #hr., #employees, #career, #entrepreneur, and #futureofwork. These hashtags represent discussions surrounding work-related stressors, job demands, leadership styles, career development, and the overall work environment. Numerous studies have highlighted the significance of work-related factors, such as high job demands, low control, and lack of support, in contributing to burnout (Demerouti et al., 2001; Maslach et al., 2001; Schaufeli et al., 2009). Effective management and leadership are crucial in reducing burnout. In this context, the hashtag #leadership ranked sixth with a total of 4,782 occurrences, followed by the related hashtags #breakfastleadership (1,615 occurrences) and #leaders (769 occurrences). The hashtag #management is in 34th place with 1,461 occurrences. According to Schaufeli and Bakker (2004), job resources provided by managers and leaders, such as support, feedback, and growth opportunities, play a vital role in preventing burnout. Bakker and Demerouti (2017) emphasize the importance of managing job demands to avoid excessive stress and exhaustion. HRM practices also play a significant role in preventing burnout. The hashtag #hr. is in 12th place with 3,223 occurrences. Research by Demerouti et al. (2001) suggests that HR practices that focus on meeting employees’ basic psychological needs, such as autonomy, competence, and relatedness, can reduce burnout. Van den Broeck et al. (2008) further emphasize the importance of HR practices that satisfy these psychological needs to enhance employee well-being and engagement while mitigating burnout. Career development is another essential aspect. The hashtag #career is in 36th place with 1,365 occurrences. Employees who actively shape their work tasks and career paths to align with their strengths and interests experience lower levels of burnout (Tims et al., 2013). It suggests that organizations should provide opportunities for employees to engage in job crafting and support their career development to prevent burnout and enhance job satisfaction. Burnout risks are also relevant in the context of the future of work (#futureofwork with 827 occurrences). Darouei and Pluut (2021) discuss the challenges of balancing the demands of virtual work and family responsibilities. The study highlights the importance of organizations implementing policies and practices that support work-life balance to prevent burnout among remote employees (Darouei and Pluut, 2021). Demerouti et al. (2012) emphasize the need to consider the work-family interface from a life and career perspective, suggesting that organizations should provide support and flexibility to address employees’ demands and challenges in managing work and family responsibilities.

Telecommuting subgroup is represented by the hashtags like #wfh, #remotework, and #workfromhome. The hashtags #wfh ranked sixth with 1,929 occurrences, #remotework 32nd with 1,479 occurrences and #workfromhome 63rd with 914 occurrences. The COVID-19 pandemic has significantly increased the prevalence of remote work, and discussions around the challenges and potential burnout risks associated with telecommuting have emerged. Research has shown that telecommuting can lead to blurred boundaries between work and personal life, increased workload, and reduced social support, which may contribute to burnout (Derks et al., 2016; Darouei and Pluut, 2021). Remote work involves working outside the traditional office, typically from home or other remote locations. While remote work offers flexibility and autonomy, it also presents challenges that can impact employee well-being and increase the risk of burnout. Derks et al. (2016) found that remote employees often face difficulties maintaining work-life balance due to blurring boundaries between work and personal life. Furthermore, the increased workload associated with remote work can contribute to burnout. Darouei and Pluut (2021) suggest that remote employees may experience higher job demands, such as increased workload and a lack of clear boundaries between work and personal life, leading to heightened stress and exhaustion. Additionally, Derks et al. (2016) found that remote employees reported lower levels of social support from colleagues than those working in traditional office settings. The absence of informal interactions and reduced opportunities for face-to-face communication may limit the availability of social support networks, which can act as protective factors against burnout.

Personal development and well-being are crucial aspects of organizational factors related to burnout. The hashtags such as #burnoutprevention, #employeewellbeing, #stressmanagement, and #coaching reflect the focus on organizational initiatives to prevent burnout and promote well-being. Proactive interventions such as stress management programs, employee support systems, and coaching effectively reduce burnout and enhance well-being (Fothergill et al., 2004; IsHak et al., 2009; Siu et al., 2014; Grawitch et al., 2015). The hashtag #stressmanagement is in 26th place with 1,747 occurrences. Stress management, for example, provides employees with techniques and skills to effectively manage and reduce stress levels, thereby mitigating the risk of burnout. The hashtag #burnoutprevention is in 48th place with 1,085 occurrences. Burnout prevention strategies aim to equip individuals with the tools and resources necessary to manage and cope with the demands of their work. Fothergill et al. (2004) highlight the importance of such interventions in enhancing well-being and preventing burnout. Employee well-being initiatives, encompassing various aspects such as physical health, mental well-being, and work-life balance, also play a critical role in burnout prevention. Implementing employee support systems that provide resources, guidance, and assistance to employees in times of stress or difficulty can contribute to a healthier work environment and reduce the likelihood of burnout. Grawitch et al. (2015) emphasize the positive impact of supportive organizational systems in fostering employee well-being and preventing burnout. Another approach to addressing burnout and promoting personal development is coaching. The hashtag #coaching is in 41st place with 1,219 occurrences. It focuses on supporting individuals in identifying and achieving their goals, enhancing their strengths, and developing strategies to manage challenges and stressors. Such interventions have been shown to reduce burnout and enhance well-being by improving self-awareness, resilience, and adaptive coping mechanisms (Grant et al., 2009).

Lastly, the subgroup of generational focus is represented by the hashtag #millennials, ranked in 33rd place with 1,465 occurrences. Discussions surrounding millennials and burnout shed light on the unique challenges faced by this generation in the workplace. Factors such as job insecurity, high expectations, and the pressure to achieve a work-life balance contribute to higher levels of burnout among millennials (Schonfeld and Bianchi, 2016). Millennials, born between the early 1980s and mid-1990s (McCrindle, 2011), have been found to experience higher levels of burnout compared to other generational cohorts. Schonfeld and Bianchi (2016) suggest that millennials face significant challenges in the modern work environment, including economic uncertainty, demanding workloads, and a strong desire for work-life balance. A study by Twenge et al. (2010) found that millennials reported higher levels of job insecurity than previous generations, which can negatively impact their well-being and increase the risk of burnout. Additionally, the high expectations placed on millennials, both internally and externally, can contribute to burnout. These expectations, combined with intense competition and the pressure to excel, can result in heightened stress levels and burnout (Twenge et al., 2012). Understanding the generational dynamics and their implications is crucial for effectively addressing burnout among different generations of employees. Organizations should consider implementing strategies that support work-life balance, provide opportunities for growth and development, and offer meaningful work experiences to help mitigate the risk of burnout.

In conclusion, work-related, telecommuting, personal development and well-being, and generational focus factors significantly impact employee burnout. While these groups are initially derived from the Twitter hashtags, it is worth noting that these categories align with research findings, substantiating their significance concerning burnout. Effective management and leadership, supportive HR practices, attention to career development, and adapting to the future of work are essential in preventing burnout and promoting employee well-being. Organizations should be mindful of telecommuting challenges and take proactive measures to support remote employees in managing their workloads, establishing clear boundaries, and fostering social connections to mitigate the risk of burnout. Further, personal development and well-being initiatives are integral to organizational efforts to prevent employee burnout. Also, addressing diverse generational differences and needs by organizations can create a supportive and engaging work environment. By understanding and addressing identified organizational factors, organizations can create healthier work environments and mitigate the risks of burnout.

The *Environmental factors* encompass many aspects associated with burnout syndrome (see Table 6 with the 22 hashtags). Notably, a significant representation of the hashtags related to the COVID-19 pandemic is observed, indicating its negative impact on the prevalence of burnout syndrome in various professions (Torres et al., 2023). The hashtag #covid19 ranks 9th with a frequency of 3,818 occurrences, making it one of the prominent hashtags within the environmental factors category. Additionally, #pandemic appears in 25th place with a frequency of 1,772, and #covid ranks 61st with 939 occurrences. The COVID-19 pandemic has brought about numerous changes in healthcare systems, resulting in reduced accessibility and increased pressures on healthcare professionals who face the challenges of an overwhelmed system (Shapiro et al., 2019). Research has also demonstrated a rise in burnout syndrome among college students during the COVID-19 pandemic as they grappled with prolonged crises and new threats (Tomaszek and Muchacka-Cymerman, 2022). The hashtag #life appears in the 77th position with a frequency of 691. At the same time, #culture emerges in the 78th place with 995 occurrences, indicating that the Twitter users recognize the significance of health and quality of life (Jois et al., 2018) and address burnout syndrome in the context of natural healing and medicine (Jones et al., 2007).

Further, the impact of the COVID-19 pandemic has heightened concerns for individuals’ health and life. The hashtag #healthcare ranks 5th with 5,131 occurrences, while #mentalhealthcare appears in the 42nd position with 1,205 occurrences, emphasizing the association between healthcare, mental health, and burnout syndrome (West et al., 2022). Burnout syndrome has been examined across various groups of employees in studies (Tzu et al., 2017), and its communication is linked to the hashtags #healing (ranked 24th) and #naturalhealing (ranked 35th), reflecting both Western and Eastern medicine perspectives (Tzu et al., 2017). Burnout syndrome has been described as impacting society’s personal, organizational, social, and economic levels (Jois et al., 2018). Several posts include the hashtags related to healthcare professions and medicine, such as #doctors (ranked 68th) with 814 occurrences, #nurses (ranked 47th) with 68 occurrences, #psychiatrist (ranked 20th) with 1,885 occurrences, #medicine (ranked 43rd) with 1,195 occurrences, #meded (ranked 46th) with 1,128 occurrences, and #recovery (ranked 28th) with 1,694 occurrences. As health and social professions face the challenges of increased patient demands, the rise in burnout syndrome becomes evident (Klein et al., 2020).

The prevalence of the hashtags in posts related to burnout syndrome indicates a connection between the health and work domains, highlighting the impact of personal and work-related stress and its management (Letson et al., 2020). De Diego-Cordero et al. (2022) state that nurses and physicians are the most at-risk groups. Medical facility management faces the challenge of addressing burnout syndrome in these high-risk employees (Chaput et al., 2015; Bernez et al., 2018). Setting up regular meetings and facilitating discussions with other professionals has been identified as one strategy to combat burnout syndrome (Chaput et al., 2015; Rosada et al., 2015). Collective sharing of group projects has also emerged to combat burnout syndrome (Torres et al., 2023). Burnout syndrome has also increased in other professional groups, such as teachers (Anama-Green, 2022), childcare center employees with multidisciplinary training and social employees (Letson et al., 2020). In their study, Suyi et al. (2017) demonstrated a link between burnout syndrome, healthcare, and staff turnover.

The prevalence of the hashtags associated with burnout syndrome suggests that the Twitter users are aware of the influence of environmental factors on their work and personal lives. Company management should acknowledge the need to address the manifestations of burnout syndrome, as it impacts the quality of work life and employee well-being, which are essential for the sustainability of organizations (Golonka et al., 2019).

The topic analysis enables a better understanding of the overall communication dynamics by identifying the connections between the hashtags. The Table 7 presents the results of the topic analysis, identifying the topics (cluster names) within the Twitter communication related to burnout syndrome from January 1st, 2019, to July 31st, 2022. A total of seven main topics were identified: (1) Workplace, (2) Healthcare, (3) Communication, (4) Stress and Therapy, (5) Rhythms of Rest, (6) Symptoms, and (7) Leadership. These topics are discussed in detail below.

**Table 7:** Results of the topic analysis.

Cluster name	Key terms	Selection count	Percentage
(1) Workplace	employee, work, workplace, job, HR, employer, company, worklifebalance, career	28,470	15%
(2) Healthcare	physician, healthcare, doctor, nurse, patient, care, medicine, health, hospital, health care, clinical	22,177	12%
(3) Communication	podcast, episode, book, youtube, listen, video, join, talk, share, live, free	20,545	11%
(4) Stress and Therapy	depression, stress, anxiety, psychotherapy, mentalhealth, psychiatrie, mindfulness, resilience, meditation, howfightdepression, notjustmood	17,239	9%
(5) Resting	day, time, sleep, week, rest, holiday, break, weekend, tired, feel	16,089	8%
(6) Symptoms	sign, burn, symptom, feel, avoid, prevent, exhaustion, signs of burnout, experience, feeling, stress	15,666	8%
(7) Leadership	leadership, team, leader, manager, coach, CEO, HR, management, coaching, executive, lead	9,538	5%

Source: Own elaboration based on collected data from the Twitter (2023).

The largest identified topic relates to the *Workplace*, accounting for 15% of the total share and occurring 28,470 times. The Workplace topic encompasses aspects of the organization, including employee, work, workplace, job, HR, employer, company, and work-life balance. The cluster focused on workplace issues mainly consists of terms related to the organizational factors of burnout, such as employee, work, workplace, HR, and career. However, the term employer is not included in any burnout factors, although there is a connection to the term entrepreneur. According to the Cambridge Dictionary (2023b), an entrepreneur is “*a person who attempts to make a profit by starting a company or by operating alone in the business world,*” while an employer is “*a person, company, or organization that employs people*” (Cambridge Dictionary, 2023a). Additionally, the term work-life balance was categorized under individual factors of burnout. However, according to Sirgy and Lee (2018), the work-life balance model is based on personal and organizational predictors. Furthermore, one of the results of their model are work-related outcomes, which are connected to performance management and HRM topics.

The second most identified topic is *Healthcare*, occurring 22,177 times and accounting for an overall share of 12%. The Healthcare topic includes aspects related to physicians, healthcare, doctor, nurse, patient, care, medicine, health, hospital, healthcare, and clinical. It mainly focuses on environmental factors of burnout (physician, doctor, nurse, healthcare, medicine). However, the term patient is not included in any specific factors. It is related to the type of burnout, specifically client-related and professional burnout. The concept of a client varies depending on the context of client-related burnout and the level of burnout. According to Chin et al. (2018), the term client refers to people who receive services from individuals providing the service. Therefore, many studies explore healthcare providers, the quality of healthcare, and the prevention of burnout (Salyers et al., 2017). However, one of the main factors influencing the topic of burnout was the COVID-19 pandemic, during which people became increasingly concerned about their healthcare. As a result, burnout research was primarily associated with healthcare providers.

*Communication* is the third identified topic, with 20,547 occurrences and a total share of 11%. This topic encompasses aspects related to different means or forms of communication, such as podcasts, episodes, books, YouTube, listening, videos, joining, talking, sharing, live events, and free content. So, there are mainly mentioned new media focused on interaction. Thus, Charoensukmongkol’s (2016) study suggests that using social media during work tends to increase burnout in employees with a low mindfulness level. Still, it lowers burnout in employees with a high mindfulness level. In addition, social media such as the Facebook, Twitter, and Instagram have played key roles in online communications in recent years. However, Han (2018) points out social media burnout concerning burnout syndrome, where he finds that ambivalence, emotional exhaustion, and depersonalization can significantly negatively influence a user’s social media continuance.

The fourth topic, *Stress and Therapy*, encompasses stress-related aspects of burnout syndrome and terms related to individual burnout factors. It holds an overall share of 9% with a total frequency of the 17,239 hashtags. Notable aspects highlight the link between burnout syndrome and stress and mental state, including terms such as depression, stress, anxiety, psychotherapy, mental health, psychiatry, mindfulness, resilience, and meditation. Terms howfightdepression and notjustmood are not included in any classification. The results show a need to prevent burnout focused on employees’ self-initiative actions (Otto et al., 2021).

The fifth topic is *Resting*, with a total share of 8% and a frequency of the 16,089 hashtags. It encompasses aspects of life’s daily rhythm and time component, including concepts like day, time, sleep, week, rest, holiday, break, and weekend. These terms are more related to personal factors of burnout. The discrepancy might be caused by employees’ working time, flexibility, and satisfaction, as stated by Lee and Chang (2022). Thus, in the context of burnout syndrome review focused on clinical physicians was carried out. The results showed that flexible arrangements concerning working time might reduce burnout risk, and improve overall job satisfaction, as exhibited by physicians working within hospital settings (Helmig et al., 2010).

The sixth topic, *Symptoms*, comprises the 15,666 hashtags, accounting for a total share of 8%. It encompasses items such as signs, burn, symptom, feel, avoid, prevent, exhaustion, signs of burnout, experience, feeling, and stress. These terms indicate various aspects associated with burnout syndrome. It is a mixture of individual factors of burnout and burnout prevention elements. Burnout symptoms, defined as consequences of chronic work stress, are an increasing problem (Aust et al., 2022).

The final topic is *Leadership*, with a total of the 9,538 hashtags, accounting for a share of 5%. It encompasses terms such as leadership, team, leader, manager, coach, CEO, HR, management, coaching, executive, and lead. It deals with HR, the management board and the team. Although this topic is the last, the importance of leadership was confirmed by Tian and Guo (2022), who explored the underlying mechanisms and boundary conditions of the relationship between transformational leadership and teacher burnout.

**Figure 3:**
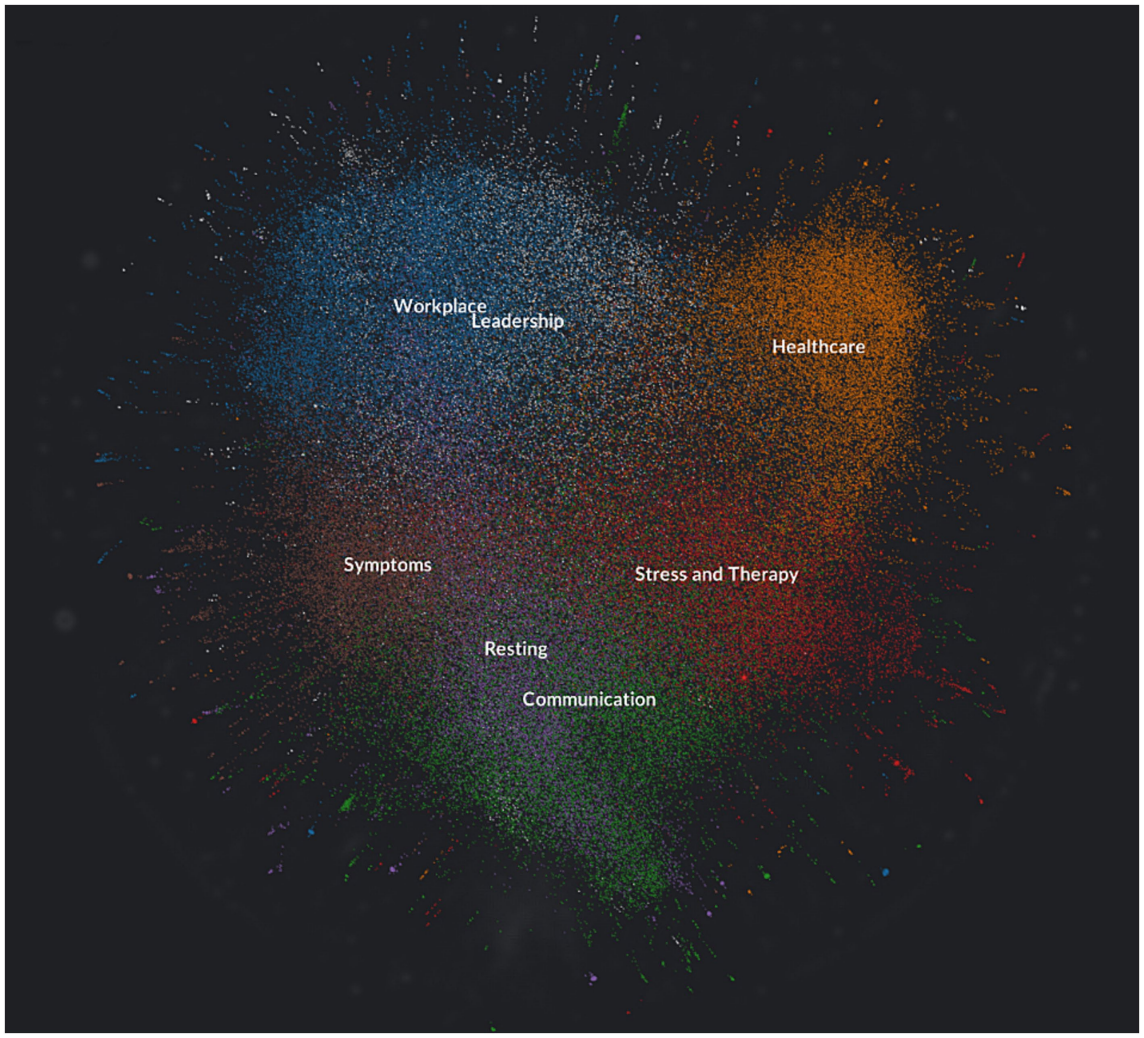
Visual analysis of theme connections and focus areas, Source: Own elaboration based on collected data from the Twitter using Graphext software (Graphext, 2023).

A visual analysis (see the Figure 3) has identified that the density of connections is evident for the themes of Resting and Communication and Workplace and Leadership. However, the theme of Resting appears to connect Communication and the Workplace with Leadership. The close link between the Workplace and Leadership themes stems from their organizational nature. Thus, the theme of Resting as a form of prevention of burnout syndrome is the focus of the education or awareness disseminated through multimedia communication (podcasts, videos, book, youtube, etc.). Topic Healthcare appears relatively isolated from other topics, probably due to its focus on the medical field. Between the Healthcare and Communication poles is the Stress and Therapy theme, intertwined with Symptoms through Resting.

## 5. Conclusion

The study results reveal that individuals discussing burnout syndrome on the Twitter perceive its symptoms as belonging to either the individual spectrum or the broader environmental factors of their lives. These findings align with the theories (Hallsten et al., 2011), establishing a clear connection between burnout syndrome and an individual’s work life. Consequently, organizations must address burnout prevention within their HRM practices, as they can take the initial steps to mitigate it. HRM can specifically address the employee’s bio-socio-psycho components, including employee care, training, planning, and evaluation. Furthermore, this employee care should be integrated into the organization’s culture, particularly regarding workplace relationships, negotiations, communication, and conflict resolution. The paper also demonstrates that discussions on burnout syndrome on the Twitter primarily revolve around stress, mental health, and self-care. It is worth mentioning the relevance of well-being and healthcare in this context. Although these topics are associated with individual factors, HRM possesses the capacity to influence these areas. The Twitter users also perceive a connection between burnout syndrome, organizational leadership, and the general environment, with the COVID-19 pandemic exerting a notable influence. Consequently, the Twitter users recognize various aspects that impact their personal lives, with a foundation in their working lives. These findings suggest that HRM should prioritize contemporary issues such as work-life balance and employee care. As demonstrated by the results, communication about burnout syndrome encompasses all its factors and should be considered from this perspective across different HRM domains.

The staff training aspect should prioritize the prevention of burnout syndrome, mental hygiene, stress management, and time management. The findings indicate that these topics are directly linked to the hashtags associated with burnout syndrome. Regarding workforce planning, it is crucial to adopt an HRM perspective that considers all social roles of employees to ensure a healthy work-life balance. The hashtags such as #work, #career, #leadership, #productivity, and #motivation demonstrate the connection between perceived burnout symptoms and their manifestation in the work environment. Employee deployment should be carefully considered when planning the workforce, including horizontal or vertical movement, to align with their work-life balance and motivation. Workforce development plans can serve as a motivational tool, but they may also create pressure to perform, potentially contributing to burnout syndrome, even if they are met. Therefore, these plans must align with the organization’s strategic objectives and the individual employees’ preferences. Regular self-development, which may involve stress management training, should also be prioritized.

The impact of burnout not only affects employee and organizational performance, but studies also highlight the factors contributing to low individual and organizational performance (Chowdhury, 2018; Firdaus et al., 2023; Wulantika et al., 2023). Specifically, individual factors such as #stress, #productivity, #resilience, #success, and #fatigue are discussed concerning the employee. On the employer’s side, organizational factors such as #hr., #work, #workplace, and #remotework are emphasized. Performance appraisal plays a crucial role in performance management. The outcomes of a formal performance appraisal system can be utilized in other areas of HRM within the Michigan model framework (Fombrun et al., 1984), such as learning and development and rewarding. Furthermore, as a core performance appraisal method, the performance appraisal interview provides valuable feedback to employees and employers.

Our study also highlights workplace relationships’ significance in preventing burnout. In addition to performance, the level of teamwork is recognized as a manifestation and impact of burnout. The most frequently mentioned the hashtags related to this topic were those related to organizational factors such as #leadership, #hr., #millenials, and #management. The hashtags like #wellbeing and #worklifebalance were the most cited concerning individual factors. These findings reinforce the importance of establishing an effective HRM system to prevent burnout syndrome, particularly in HRM, performance appraisal, training and development, workforce planning, and employee well-being.

One factor significantly impacting employee burnout is organizational culture, which necessitates special attention from HRM and decision-makers (Ghorbanian et al., 2018). While burnout syndrome is often seen as an individual issue, the responsibility for its management is increasingly shifting from employees to employers. Employee care serves as a reflection of organizational culture, and therefore, proactive efforts to enhance organizational culture and prioritize employee well-being are highly recommended. Creating a work environment that promotes well-being involves various measures such as improving the ergonomics of the workspace, providing benefits that support mental and physical health, offering tailored support for parents or caregivers, facilitating training opportunities, optimizing recruitment processes, implementing effective onboarding and outplacement practices, and fostering company rituals. The significance of organizational culture’s influence is exemplified by the findings of the topic analysis, where the most prevalent identified topic is Organization, encompassing key terms such as #employee, #work, #workplace, #job, #HR, #employer, #company, #worklifebalance, and #career.

Theoretical implications of this paper offer a fresh perspective on understanding the communication surrounding burnout syndrome, highlighting the importance of individual, organizational, and environmental factors. This breakdown, encompassing these three factors, holds particular significance within organizational management theory and HRM. Additionally, the study’s theoretical contribution lies in its unique approach of utilizing big data analysis from the Twitter to investigate burnout communication, as no prior studies have explored this.

From a practical point of view, the study’s findings have implications for strategic management and HRM. At the strategic management level, organization’s can incorporate the concept of burnout syndrome into their values and philosophy, focusing on organizational aspects, working hours, and work-life balance. This approach aligns with work-life balance, well-being, and self-care principles, enhancing employee care, workplace relationships, communication, and overall job satisfaction. A satisfied and motivated workforce provides a competitive advantage for employers and helps foster and reinforce the organizational culture, values, and strategic goals. For HRM, the study findings offer practical benefits for managers in various areas. These include workforce planning, the formulation of HRM policies, team building, team leadership, employee evaluation, and motivation. Given the nature of jobs and roles, which often entail significant responsibility and require refined soft skills such as decision-making, adaptability, communication, autonomy, and stress management, managers can apply a management approach to structure job descriptions, optimize staff development plans, streamline job and role descriptions, and manage employees’ careers. This approach addresses factors associated with burnout and promotes a healthier work environment.

While this study has provided valuable insights into the communication of burnout syndrome on the Twitter and its implications for HRM, it is vital to acknowledge the limitations that may affect the generalizability and interpretation of the findings. First is the restriction to a specific location of posts, which makes it challenging to generalize the findings to other contexts. Despite this limitation, the study has provided valuable insights based on the perspectives of HRM professionals regarding burnout prevention. Second is the focus on a specific target group. However, the results offer a comprehensive understanding of the factors associated with burnout from HRM and social media perspectives. The findings have implications for burnout prevention and communication on social media, particularly in collaboration with HRM professionals. Third is the timeframe of the data collected. It would be beneficial to compare the results obtained from this study on burnout communication on the Twitter with other studies, such as an omnibus study or a panel study, to determine whether the hashtags and topics related to burnout syndrome change over time. Future studies should investigate burnout syndrome in social media communication and its relationship with HRM, organizational culture, work-life balance, and well-being. Further, future studies should address performance appraisal and outcomes to prevent burnout syndrome. By addressing these areas, further insights can be gained to advance our understanding of burnout and its management in contemporary contexts.

## Data availability statement

The datasets analyzed for this study can be found at the Zenodo: https://zenodo.org/record/8014694. In consideration of Twitter’s terms and conditions, along with ethical and legal considerations, the dataset includes Tweet IDs to ensure the reproducibility of the research.

## Author contributions

LK and LP initiated the study and contributed to data curation, formal analysis, visualization, and methodology. GJ, KD, KZ, and KK contributed to the conception, design of the study, and performed the analysis, wrote sections of the manuscript. GJ, KD, and KZ prepared parts of the text, resources, and wrote the first draft of the manuscript. KZ prepared the figures, tables, and translated and finalized the manuscript to the template. KK made conceptualization. GJ, KK, and KZ made supervision. LP funding acquisition. All authors contributed to the manuscript’s revision and read and approved the submitted version.
